# Clinical parameters of laminoplasty and laminectomy with fusion in the treatment of cervical spondylosis and analysis of postoperative sagittal balance

**DOI:** 10.55730/1300-0144.5713

**Published:** 2023-08-11

**Authors:** Mehmet Yiğit AKGÜN, Özkan ATEŞ, Mehmet Ali TEPEBAŞILI, Caner GÜNERBÜYÜK, Ali Fahir ÖZER

**Affiliations:** 1 Department of Neurosurgery, Koç University Hospital, İstanbul, Turkiye; 2 Spine Center, Koç University Hospital, İstanbul, Turkiye

**Keywords:** Cervical, lordosis, laminectomy, laminoplasty, balance

## Abstract

**Background/aim:**

Cervical spondylosis is a progressive disease that causes degenerative changes affecting the spine, intervertebral discs, facets, and ligaments. With anterior and posterior surgical interventions, effective treatments can be applied in cervical spondylotic myelopathy (CSM). The relationship between regional and global spinal alignment and functional and pain outcomes was examined and it was revealed that these parameters play a significant role in obtaining good results. The aim of this study was to compare the perioperative and follow-up results of patients with CSM who underwent laminoplasty or laminectomy with fusion.

**Materials and method:**

CSM patients who were operated on between 2015 and 2020 and had at least 2 years of clinical and radiological follow-up were analyzed retrospectively. The patients were divided into 2 groups as the laminoplasty group and the laminectomy with fusion group, according to a simple random method. Demographic, clinical, radiological, and perioperative parameters were examined. Measurements were made by an independent observer using Surgimap and 2 years was considered to be sufficient time for the spine to take its final shape.

**Result:**

A total of 112 patients, including 68 males and 44 females, were included. Of these patients, 69 were in the laminectomy with fusion group, and 43 were in the laminoplasty group. Patient ages ranged from 39 to 85 years. The mean follow-up period was 36.28 months. In both groups, at the 3-month follow-up, a statistically significant improvement in the clinical parameters (neck disability index, visual analogue scale, modified Japanese Orthopaedic Association scores) was observed. When the preoperative cervical radiological parameters were evaluated, no statistically significant difference was found between the groups. The C2–C7 lordotic angles and the cervical SVA values were increased in the postoperative period, for both groups (p < 0.001). Although it is noteworthy that the increases were higher in the laminectomy with fusion group, no statistically significant difference was found between the groups.

**Conclusion:**

Deformity in a spinal segment may indirectly affect another segment. Being aware of the compensatory mechanisms and radiological parameters will help in determining the effective treatment plan.

## 1. Introduction

Cervical spondylosis is a progressive disease that causes degenerative changes affecting the spine, intervertebral discs, facets, and ligaments. Spondylosis usually begins with degenerative changes in the intervertebral disc space and leads to secondary changes in the surrounding structures. Cervical spondylotic myelopathy (CSM) occurs due to these degenerative changes that damage the spinal cord. Neuronal dysfunction develops secondary to the inhibition of afferent and efferent nerve fibers as a result of spinal cord damage in CSM.

CSM is the most common form of spinal cord injury in adults and accounts for the majority of nontraumatic spinal cord injuries. CSM clinic is detected in approximately 10% of patients aged 55 and over. In addition, findings consistent with cervical spondylosis, which has a risk of progression, were observed in approximately 85% of the imaging performed in adults over 60 years of age. CSM is an insidious disease that does not show clinical symptoms in the early period. Early diagnosis and treatment of CSM before it causes irreversible spinal cord damage is critical to maintain quality of life.

With anterior and posterior surgical interventions, effective treatments can be applied in CSM and satisfactory results can be obtained. However, in the last 5–10 years, the relationship between regional and global spinal alignment and functional and pain outcomes has been examined and it was revealed that these parameters play a significant role in obtaining good results. The traditional posterior approaches, laminectomy with fusion and laminoplasty applications, have been discussed in the literature. However, there are shortcomings in the long-term comparative results of these methods and their effects on cervical spinal alignment.

The aim of this study was to compare the perioperative and follow-up results of patients with CSM who underwent laminoplasty or laminectomy with fusion. In addition, cervical alignments in the clinical follow-up of the patients and their relation to the postoperative findings were evaluated.

## 2. Materials and methods

In this study, all procedures performed were in accordance with the ethical standards of the institutional and national research committee and with the 1964 Helsinki Declaration and its later amendments or comparable ethical standards. Informed consent was obtained from all participants included in the study. Ethical committee approval was received from the Ethics Committee of Tekirdağ Dr. İsmail Fehmi Cumalıoğlu City Hospital (Approval No: 2023/51).

CSM patients who were operated on between 2015 and 2020 and had at least 2 years of clinical and radiological follow-up were analyzed retrospectively. The sample size was determined using G*Power (version 3.1.9.4). Reaching the required number of files according to the sample calculation was determined as the primary endpoint of the study [1, 2]. Patients with complete radiological and clinical follow-up/treatment, over 18 years of age, with no improvement in symptoms with classical analgesic treatment and conservative approaches, with typical imaging features of cervical spondylosis, and with CSM clinical symptoms were included in the study. Patients who could not accurately assess their symptoms or cooperate with diagnosis and treatment, had high comorbidities, had severe organ failure, and had a history of previous cervical surgery were excluded.

The patients were divided into 2 groups as the laminoplasty group and the laminectomy with fusion group, according to a simple random method. Expansive laminoplasty was performed on the patients with a unilateral approach using a mini plate (i.e., open-door laminoplasty). Laminectomy with fusion was performed with lateral mass screw-rod fixation. In order to achieve homogeneity in both groups and disable additional parameters that may affect spinal stability, only the patients operated on between cervical vertebrae 3 (C3)–C6 were included in the study. The selection of which surgical technique to use was considered on an individual case-by-case basis. Patients with radiculopathy secondary to unilateral foraminal compression and nonkyphotic cervical spine alignment were evaluated as candidates for laminoplasty, while patients with bilateral foraminal compression and severe neck pain secondary to CSM clinic were considered candidates for laminectomy and fusion. It was assumed that our modification of the surgical technique for laminoplasty would directly affect the results. A bony groove created using a high-speed drill approximately 0.4–0.5 mm medial (not at the junction) of the lamina and facet junction provides better postoperative results.

All of the patients were operated on by 1 or 2 senior surgeons. They had all been diagnosed via magnetic resonance imaging (MRI). Pre- and postoperative lateral standing radiographic measurements were taken using standard lateral cervical X-rays. The protocol was undertaken with the patients standing in a neutral position while looking straight ahead. Health-related quality of life (HRQOL) measures (the Short Form-36 Health Survey), including the neck disability index (NDI), visual analogue scale (VAS) for pain, and modified Japanese Orthopaedic Association (mJOA) scores, were applied to the groups. Pearson product-moment correlation coefficients were calculated between pairs of radiographic measures and HRQOL scores.

Perioperative outcomes that were collected included estimated blood loss (BL; in milliliters), complications, and hospital length of stay (LOS; date of surgery to date of discharge). Complications were defined as any unforeseen event requiring additional medical and/or surgical intervention.

The parameters examined on X-rays pre- and postoperatively were as follows: C0–C2 angle, C2–C7 lordosis, sagittal vertical axis (SVA) (C2–C7 SVA) and T1 slope. The radiological parameters and HRQOL scores were evaluated preoperatively, at 3 months, 6 months, and 2 years postoperatively by direct radiography. Measurements were made by an independent observer using Surgimap (Globus Medical Inc., Audubon, PA, USA) and 2 years was considered to be sufficient time for the spine to take its final shape (Figure).

### 2.1. Statistical analysis

The statistical analysis was performed using IBM SPSS Statistics for Windows 20.0 (IBM Corp., Armonk, NY, USA). The estimated power was 0.80, alpha (margin of error): 0.05, and the effect size was 0.4. Accordingly, the sample size was determined as 50 for the chi squared test. All files (112 files) were included in the study, as the number of files remaining after assessing all of them according to the exclusion the criteria. For the significant values, which groups were different from each other and what the source of this difference was between the groups were examined by postoperative comparison tests, including the Tukey honestly significant difference test. Since the variables in the data were obtained with a proportional or intermittent scale and were normally distributed, Pearson correlation analysis was performed. A 2-tailed p < 0.05 was considered statistically significant.

## 3. Results

A total of 112 patients, including 68 males and 44 females, were included. They were divided into 2 groups as the laminoplasty or laminectomy with fusion group according to their radiological and clinical features: 69 patients were in the laminectomy with fusion group, and 43 patients were in the laminoplasty group. Patient ages ranged from 39 to 85 years with a mean of 67.18 ± 13.73 years. The mean follow-up period was 36.28 months (range 24–120 months). Baseline demographic and procedural characteristics by localization are summarized in Table 1.

### 3.1. Clinical outcomes

In both groups (laminoplasty vs. laminectomy with fusion), at the 3-month follow-up, a statistically significant improvement in the clinical parameters (NDI, VAS, mJOA scores) was observed (p = 0.038 and p = 0.001, p = 0.14 and p = 0.001, and p = 0.21 and p = 0.001, respectively. The neurological recovery levels and reflections on the clinic were satisfactory in all of the patients.

For the laminectomy with fusion group, the difference in the clinical relief between the end of month 3 and the last clinical follow-up was not statistically significant (p > 0.05). Although a slight increase in parameters was observed in the last clinical follow-up, no significant clinical reflection was observed. The opposite was noted in the laminoplasty group, wherein the difference in the clinical relief between the end of month 3 and the last clinical follow-up was statistically significant (p < 0.05). At the end of 2 years, there was a serious regression in the axial symptoms of the patients. In both groups, it was observed that the neurological deficits recovered, and no additional pathological examination findings were detected (Table 2).

There was no statistically significant difference between the operation times of the patients and BL during the operation in either group. In addition, there was no statistically significant difference between the groups in terms of complication rates and hospital LOS (Table 3).

### 3.2. Radiological outcomes

When all of the patients were evaluated, the mean preoperative C2–C7 lordotic angle (cervical sagittal Cobb) was 11.23 ± 2.57°. The mean preoperative C2–7 lordotic angles were 10.48 ± 2.82° in the laminectomy with fusion group and 13.35 ± 3.42° in the laminoplasty group (p = 0.077). The mean preoperative cervical SVA was 12.17 ± 7.11 mm in the laminectomy with fusion group and 13.38 ± 6.97 mm in the laminoplasty group. The mean preoperative T1 slope was 24.25 ± 5.41° in the laminectomy with fusion group and 24.38 ± 4.97° in the laminoplasty group. The mean preoperative C_0_–C_2_ cobb angle was 19.54 ± 7.32° in the laminectomy with fusion group and 19.38 ± 6.83° in the laminoplasty group. When the preoperative cervical radiological parameters were evaluated, no statistically significant difference was found between the groups. Since only the patients with C3–C6 stabilization were included in the study, there was no statistically significant difference between the groups in regard to the number of segments operated on.

The radiological evaluation of the patients is given in Table 4, wherein the values preoperatively, 3 months, 6 months, and 2 years postoperatively were compared. The C2–C7 lordotic angles and the cervical SVA values increased in the postoperative period, for both groups (p < 0.001). Although it is noteworthy that the increases were higher in the laminectomy with fusion group, no statistically significant difference was found between the groups. Moreover, in the laminoplasty group, the cervical sagittal Cobb values increased more significantly in the 2-year follow-up and were correlated with the gradual decrease in neck pain.

At the last follow up, the mean postoperative C2–C7 lordotic angles were 13.48 ± 3.82° in the laminectomy with fusion group and 16.54 ± 2.97° in the laminoplasty group. The mean postoperative cervical SVA was 23.42 ± 6.52 mm in the laminectomy with fusion group and 24.49 ± 6.64 mm in the laminoplasty group. The mean postoperative T1 slope was 26.25 ± 4.38° in the laminectomy with fusion group and 25.72 ± 5.72° in the laminoplasty group. The mean postoperative C_0_–C_2_ cobb angle was 21.36 ± 6.88° in the laminectomy with fusion group and 21.45 ± 5.24° in the laminoplasty group. When the postoperative cervical radiological parameters were evaluated, no statistically significant difference was found between the groups. Considering the values of the C_0_–C_2_ cobb angle and T1 slope angle, no statistically significant change was observed in the postoperative period (p > 0.05).

Control cervical MRI was performed on the patients in the early postoperative period and at the last follow-up. Adequate compression was observed in all of the patients on the postoperative MRIs.

## 4. Discussion

Although cervical myelopathy can be treated with anterior and posterior decompression, multilevel cases are usually treated with posterior decompression due to possible complications of anterior decompression [3–5]. Considering the posterior approaches, laminoplasty and laminectomy with fusion are the most commonly used surgical techniques. In the literature, no significant superiority has been shown between these treatments. Several different surgical methods are available in these surgical techniques. In the laminoplasty group, open door laminoplasty with a unilateral approach using a mini plate was chosen, while for the laminectomy with fusion groups, lateral mass screw-rod fixation was used. Determining the best surgery for CSM differed according to many factors such as cervical lordosis, instability, foraminal compression grade, and clinical signs [6–8].

Clinical improvement (mJOA, VAS, NDI) was observed at the end of month 3 postoperatively both groups. While no significant clinical improvement was observed in the 2-year follow-up in the laminectomy with fusion group, it was observed in the laminoplasty group. Furthermore, Özer et al. [9] reached a similar conclusion in their study. It is obvious that after laminoplasty, it takes some time to reach a final clinical outcome. Therefore, it would be a good decision to inform patients that after laminoplasty, their complaints will decrease over time in the postoperative period.

Loss of cervical lordosis is closely related to neck pain [7]. CSM and other cervical pathologies that disrupt the cervical alignment can lead to this. Therefore, ideal treatment in CSM also should intent to provide cervical lordosis as well as to treat the primary pathology. A metaanalysis study suggested that cervical lordosis decreased equally in after laminoplasty and laminectomy with fusion [10]. In addition, it was reported that the T1 slope and C2–C7 SVA could be used to predict loss of cervical lordosis after laminoplasty [11]. Contrary to this, in the present study, the lordotic angles had increased in both groups. Laminectomy with fusion provided much better cervical lordosis due to posterior fixation. In parallel, it was observed that laminectomy with fusion increased the cervical C2–C7 lordotic angle more than laminoplasty but that it was not statically significant. However, this difference did not have a significant effect on the early postoperative clinical outcomes either. Therefore, laminectomy with fusion seems to be a better option than laminoplasty in patients with cervical lordosis loss prior to surgery.

Lau et al. [7] suggested that greater lordosis is associated with better pain outcomes patients who underwent laminoplasty. In the current study, significant cobb angle increasement was observed in the laminoplasty group at the 2-year follow-up compared to that early postoperatively. Furthermore, this increase was correlated with relief in neck pain. Moreover, the postoperative SVA increased in both groups. However, this increase was not significant between the groups. Kato et al. [11] reported in their retrospective study that the SVA value affects other cervical parameters and preoperative SVA values of >35mm cause poor postoperative clinical results. In addition, Mun et al. [12] stated that changes in the SVA values were higher in elderly patients.

Neck and shoulder pain after laminoplasty is a challenging consequence, which may last for years, and can be seen at rates up to 60% [13–15]. Several studies have suggested that dissection of the cervical muscles adhering to cervical vertebrates and impairment of the cervical alignment cause instability and neck pain [8, 16]. Herein, however, this pain decreased over time and the clinical and radiological parameters of the patient improved during the postoperative period. It is believed that it was due to the healing of the affected structures over time and the restoration of the cervical stability. Laminectomy with fusion provides stability in the early period and reduces neck pain due to instability. Therefore, laminectomy with fusion may be the best treatment option in patients with preoperative neck pain due to cervical instability concomitant to CSM.

Although, neck pain due to instability is one of the most important postoperative problems of laminoplasty, laminectomy with fusion is generally related with higher morbidity due to possibility of nonunion, instrument failure, and adjacent segment degeneration. [8, 17–19]. The material used and the experience of the surgeon may be a factor in reducing complications such as nonunion and instrument failure. Posterior fusion reduces range of motion and causes adjacent segment degeneration, which is one of the main problems of laminectomy with fusion. Thus, this may lead to degeneration related to radiculopathy and myelopathy.

The operative time and BL may be determining factors in choosing the treatment method in patients with multiple comorbidities. It has been reported that the mean operative time and BL are higher in laminectomy with fusion than in laminoplasty [20–22]. Herein, there was no statistically significant difference between the operation times, the BL during the operation, and the hospital LOS. Differences in the surgical technique and instruments used may have caused this difference between the literature results and those in the present study.

It was seen that the number of samples in the current study was higher when compared to the literature. While it was observed that the clinical follow-up period of the studies was 2 years on average, herein, the average clinical results were obtained in 3 years. In addition, it was stated that a 2-year period was required for the spine to take its final shape, which has not been mentioned in the literature before. It was clearly emphasized that a significant improvement was observed in both the lordotic angle and clinical parameters after 2 years, especially in the laminoplasty group. In the early period, it was observed that laminectomy with fusion increased the cervical C2–C7 lordotic angle more than laminoplasty did, but that it was not statically significant. Therefore, in the present study, neck pain and cervical parameters in the preoperative period played a key role in choosing the surgical method. Finally, another issue that has not been clearly emphasized in the literature is the surgical approach. We attribute the results in the early clinical follow-up of the laminoplasty patients to our surgical approach, which is relatively comfortable compared to the literature. A bone groove created using a high-speed drill approximately 0.4–0.5 mm medial (not at the junction) of the lamina and facet junction provided better postoperative results.

The retrospective design of this study was one of the most important limitations. Prospective randomized controlled trials and sample enlargement will yield more valuable results. The follow-up period of 3 years was relatively short, because it is necessary to have an average of 5 years of follow-up in order to see the final state of the spine. The number of patients was much smaller in the laminoplasty group than in the laminectomy with fusion group. Since this study was retrospective in nature, the effects of thoracolumbar and spinopelvic parameters on postoperative changes of the cervical sagittal alignment were not examined using radiographs of the whole spine.

## 5. Conclusion

Spinal radiological parameters and clinical findings should be evaluated together as a whole. Deformity in a spinal segment may indirectly affect another segment. Being aware of the compensatory mechanisms and radiological parameters will help in determining the effective treatment plan. It can be concluded that, with appropriate patient selection in the treatment of CSM, both laminectomy with fusion and laminoplasty seem to be clinically beneficial to patients.

## Figures and Tables

**Figure:**
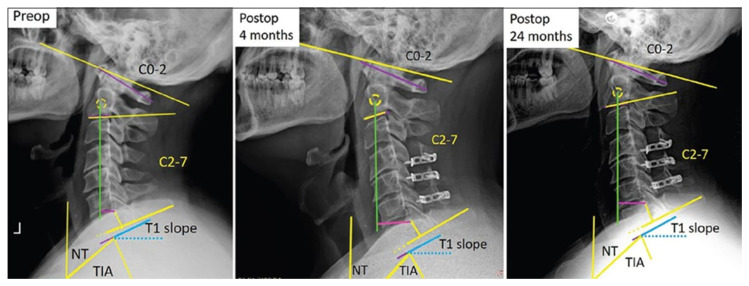
Measurement parameters in the patient who underwent laminoplasty.

**Table 1:** Demographic and procedural characteristics of the patients.

Variables	
Age, (years)	67.18 ± 13.73
Male/female	68/44
Operation (%)	
Laminectomy with fusion Laminoplasty	69 (61.6%)
	43 (38.4%)

**Table 2:** Clinical parameters of the patients.

Clinical parameters	Preoperatively	3 months	6 months	2 years	p-value
Laminectomy with fusion					
VAS	8.41 ± 2.34	6.94 ± 2.13	5.15 ± 1.98	4.36 *± 2.41*	<0.001
mJOA	10.17 ± 1.45	13.12 ± 1.87	14.96 ± 0.76	15.98 *± 1.63*	<0.001
NDI	38.41 ± 2.36	25.12 ± 2.01	25.43 ± 1.97	26.25 *± 1.43*	<0.001
Laminoplasty					
VAS	8.92 ± 2.41	7.34 ± 2.65	6.32 *± 2.13*	4.98 *± 2.76*	<0.001
mJOA	9.43 *± 1.87*	12.54 ± 1.65	13.87 *± 2.1*	15.34 *± 1.65*	<0.001
NDI	33.25 *± 2.21*	25.11 ± 2.54	25.32 *± 2.22*	25.72 *± 1.76*	<0.001

Data are given as the mean *±* standard deviation

**Table 3:** Perioperative outcomes of the patients.

Perioperative outcomes	Laminectomy with fusion	Laminoplasty	p-value
Mean operating time (min)	132.48 *± 4.32*	143.23 *± 4.87*	0.454
Estimated BL (mL)	302.54 *± 5.21*	309.43 *± 4.93*	0.501
Complication rates (%)	6	7	0.054
Mean LOS (days)	3.7 *± 2.44*	3.1 *± 2.21*	0.512

**Table 4:** Radiological parameters of the patients.

Radiological parameters	Preoperatively	3 months	6 months	2 years	p-value
Laminectomy with fusion					
C2–C7 lordotic angle	10.48 ± 2.82	12.27 ± 1.67	12.53 ± 0.69	13.48 *± 3.82*	<0.001
Cervical SVA	12.17 ± 7.11	21.56 ± 2.91	22.65 ± 1.67	23.42 *± 6.52*	<0.001
T1 slope	24.25 ± 5.41	25.12 ± 4.74	25.43 ± 2.65	26.25 *± 4.38*	>0.05
C_0_–C_2_ Cobb angle	19.54 ± 7.32	20.87 ± 3.45	21.12 *± 1.76*	21.36 *± 6.88*	>0.05
Laminoplasty					
C2–C7 lordotic angle	13.35 ± 3.42	15.14 ± 1.94	15.34 *± 1.32*	16.54 *± 2.97*	<0.001
Cervical SVA	13.38 *± 6.97*	21.43 ± 5.97	23.56 *± 6.14*	24.49 *± 6.64*	<0.001
T1 slope	24.38 *± 4.97*	25.11 ± 4.21	25.32 *± 4.97*	25.72 *± 5.72*	>0.05
C_0_–C_2_ Cobb angle	19.38 *± 6.83*	20.43 ± 6.23	20.97 *± 5.98*	21.45 *± 5.24*	>0.05
